# Stochastic partial budget analysis of strategies to reduce the prevalence of lung lesions in finishing pigs at slaughter

**DOI:** 10.3389/fvets.2022.957975

**Published:** 2022-10-14

**Authors:** Josefine Jerlström, Wei Huang, Carl-Johan Ehlorsson, Ingvar Eriksson, Amanda Reneby, Arianna Comin

**Affiliations:** ^1^Department of Animal Environment and Health, Swedish University of Agricultural Sciences, Uppsala, Sweden; ^2^Department of Economics, Swedish University of Agricultural Sciences, Uppsala, Sweden; ^3^Farm and Animal Health Association, Staffanstorp, Sweden; ^4^Department of Disease Control and Epidemiology, Swedish National Veterinary Institute, Uppsala, Sweden

**Keywords:** animal health, profitability, porcine, farrow-to-finish farms, meat inspection, pleurisy

## Abstract

Ante- and post-mortem inspections of food-producing animals at slaughter are mandatory activities carried out in many countries to ensure public health, animal health, and meat quality. In finishing pigs, lung lesions are the most frequent defects found in meat inspections. It is possible to implement managerial strategies on-farm to reduce the occurrence and spread of respiratory diseases, but such strategies come with additional costs that could impede implementation. This study assessed the economic impact of two strategies aimed at reducing the prevalence of lung lesions in finishing pigs at slaughter by improving the health conditions of the animals during the production cycle. First, a farrow-to-finish pig farm with 355 sows was modeled based on the current standard practice for finishing pig production in Sweden, using economic data, meat inspection data and biological variables from the literature and expert opinions. A partial budget analysis was then performed in which the baseline farm was compared with two hypothetical strategies aimed at reducing the occurrence and spread of respiratory diseases during pig production: (S1) avoiding mixing of litters after weaning and (S2) keeping purchased pregnant gilts separated from sows during gestation, farrowing and lactation. Both these strategies intended to reduce the occurrence of respiratory disease in finishing pigs at slaughter gave an average gain in annual net income (33,805 SEK in S1 and 173,160 SEK in S2, equal to 3,146€ and 16,113€, respectively, at the time of analysis), indicating that both were economically sustainable under the assumed conditions. The impact analysis of the two strategies revealed that the reduced prevalence of lung lesions when adopting one of the strategies was the most influential factor in net benefit change on the farm. Overall, the results suggest that with the increasing prevalence of lung lesions in Swedish pig production (as also observed worldwide in recent years), adopting an effective strategy to decrease respiratory infections will become more relevant and economically beneficial.

## Introduction

For decades, respiratory diseases have been the most important health problem in the global pig production industry (1). They also involve a large economic burden leading to losses for the producer, as pigs infected with respiratory pathogens are associated with an increased mortality rate (2), higher veterinary and medication costs, reduced average daily weight gain during the grower-finisher period (3, 4) and reduced feed conversion efficiency (5, 6). In addition, respiratory disease impairs pig welfare, since it affects health status and biological functioning, and thus causes distress in the animal (7).

In the European Union (EU), ante- and post-mortem inspections of food-producing animals are mandatory and encompass animal health surveillance, protection of public health and ensuring meat quality (8). Post-mortem meat inspection provides valuable indicators for monitoring the effect of disease control measures and estimating animal health status on-farm. Lung lesions are the most frequent defect found at meat inspection of slaughter pigs worldwide (9). Some of these lesions are indicative of specific pathogens, while others simply indicate a specific respiratory disease. Detection of pathological lung lesions during post-mortem inspection can lead to carcase devaluation and partial or whole carcase condemnation, which results in direct economic losses for primary producers. In addition, it can negatively affect slaughter operations by reducing slaughter line speed and increasing the labor required to handle the carcases, causing further economic losses.

The incidence of pleurisy and pneumonia at slaughter has increased globally in the past two decades (10) and the high prevalence of lung lesions and associated negative impacts on farm economics are a known issue (11). Since the prevalence of lung lesions in pigs depends on both infectious agents and environmental factors [e.g., air quality, production system, flooring type, number of pigs per pen and direct contact (nose-to-nose) between infected and susceptible pigs (11)], strategies to improve animal health, working environment and farm finances need to be implemented. Vaccination has been used to control pleurisy and lung lesions due to *Mycoplasma hyopneumoniae* (Mhyo) and *Actinobacillus pleuropneumoniae* (App). However, vaccination without proper hygiene management appears to be economically inefficient on farms with a high prevalence of App (12). In addition, appropriate gilt acclimatization strategies are crucial, since piglets tend to be infected with respiratory pathogens during farrowing or lactation (2, 11, 13). Regardless of the nature of the infection, it is possible to implement managerial strategies that reduce the occurrence and spread of respiratory diseases in finishing pigs. However, such strategies come with additional costs that may impede their implementation.

In this study, the costs of two such managerial strategies were assessed using partial budget analysis, a planning and decision-making tool that allows the evaluation of whether a new strategy in farm management or production practices will change the net benefit by considering the effects on net cost change and net benefit change (14–16). Stochastic partial budget analysis, where the range of values and the distribution of a selected indicator are considered, has become a popular tool for analyzing the economic effects of animal disease control (17, 18) and for decision-making to improve animal welfare (19). Measuring and estimating animal health and welfare in terms of economics is complex due to the multifactorial background and the limitations in the assumptions needed (20). However, economic research is important for the animal industry due to low-profit margins and for policymakers planning incentives to encourage animal welfare and health improvements.

The overall aim of this study was to assess the economic impact of two strategies aimed at reducing the prevalence of lung lesions detected at meat inspection of pigs by improving the health conditions of the animals during the production cycle. Two strategies that are currently recommended to Swedish pig producers to decrease the prevalence of lung lesions were considered: (i) avoiding mixing litters after weaning (S1) (21), and (ii) keeping purchased pregnant gilts separated from sows during gestation, farrowing and lactation (S2) (22). Stochastic partial budget analysis was used to measure how these two strategies affected the associated cost and net benefit change in finishing pig production.

## Materials and methods

### Baseline farm model

A farrow-to-finish farm with 355 sows (i.e., the average number recorded in 2018 and the reference year) was modeled using economic data from the WinPig^®^ farm management software (23), and the meat inspection data were collected from Swedish abattoirs in 2018 and biological parameters were based on the literature. Additionally, personal communications from experts at the Farm and Animal Health Association (knowledgeable in pig production, disease control and economics) validated the baseline farm and supplied relevant information. Farrow-to-finish farms are integrated production systems that keep both nursery and finishing pigs and include all phases of the pig's life cycle, i.e., breeding, gestation, farrowing, lactation, weaning and growing the pig to a finishing weight of approximately 120 kg (24, 25). However, replacement sows are commonly recruited as 7-week pregnant gilts from the nucleus or multiplying herds. The total production period for adult sows lasts around 10 months: 4 months for breeding and gestation and six months for rearing the litter born to market weight. In the modeled farm, piglets were assumed to be crossbreeds between Landrace/Yorkshire bred with Duroc or Hampshire.

The baseline farm model was built in Microsoft Excel 2016 (Microsoft Corp., Redmond, WA, USA) and based on current standard practices in finishing pig production in Sweden (Table 1). Since a farrow-to-finish production system was modeled, two sub-models were needed, one for the finishing phase and one for the gestation, farrowing, suckling and growth phases. Detailed figures about farrow-to-finish pig production are reported in Supplementary material 1.

**Table 1:** Summary of variables included in the baseline farm model. Costs shown are associated with the finishing phase of production on a yearly basis.

**Variable**	**Mean value**	**Source**
Number of finishing pigs sold	9,308	Meat inspection data 2018
Production days	97	WinPig 2018 (23)
Farm mortality (%)	1.8	WinPig 2018
Average production days for animals dead before slaughter	67.9	Expert opinion
Average daily feed intake (MJ NE)	26.4	WinPig 2018
Feed costs (SEK/MJ NE)	0.264	WinPig 2018
Working hours per pig per day	0.25	WinPig 2018
Labor costs (SEK/hour)	275	WinPig 2018
Cost of medicines, treatments and vet visits (SEK/pig)	5	WinPig 2018
Cost of carcass disposal (SEK/pig)	524	Svensk lantbrukstjänst, 2018 (26)
Prevalence of lung lesions at meat inspection (%)	19.2	Meat inspection data 2018
Average carcase weight for pigs without lung lesions (kg)	93.2	Meat inspection data 2018
Average carcase weight for pigs with lung lesions (kg)	92.3	Meat inspection data 2018
Meat sale price (SEK/kg)	18.58	KLS Ugglarps 2018 (27)
Deduction for lung lesions at meat inspection (SEK/pig)	−20	Personal communication
Loss of value due to carcase condition* (SEK/kg)	−0.30	KLS Ugglarps 2018

^*^It was assumed that carcases with lesions were allocated to a lower classification score (due to poorer general condition) and thus suffered a price penalty (28).

The prevalence of lung lesions was estimated from data on pathological findings recorded at meat inspection of finishing pigs slaughtered in 2018 in the 10 largest abattoirs in Sweden. Upon meat inspection, up to five different lesions can be recorded at the carcase level using a standardized coding system (29). To match the baseline farm model, only farms that slaughtered between 7,000 and 12,000 pigs, which was the expected annual production of a farrow-to-finish farm with 355 sows, were considered. In total, 468,774 carcases from 53 different farms were represented in the data. Variables in the baseline farm model used in the partial budget analysis are listed in Table 1. The partial budget analysis was based on data for the finishing phase of production since this was assumed to have the greatest economic impact. Variables were assumed to have a normal distribution, with a 10% standard deviation (sd).

### Strategies to reduce the prevalence of lung lesions

In scenario S1, it was assumed that the producer avoided mixing litters after weaning. In conventional integrated pig production, piglets are commonly mixed after weaning to ensure even groups of animals of approximately similar body weight. While this procedure allows the available space to be maximized, it also promotes the spread of infections, since pigs from different litters have differences in their immune system and bacterial flora. According to previous studies, avoiding mixing litters and rearing pigs in sibling groups seems to increase their performance, resulting in decreased detection of lung lesions at meat inspection (21). Table 2 summarizes the additional variables included in the partial budget analysis of model S1.

**Table 2:** Additional variables used in the partial budget analysis model for strategy S1 (avoiding mixing litters).

**Variable**	**Mean value**
Reduction in finishing pigs sold due to non-maximization of available space (%)	−2.5
Farm mortality (%)	0.9
Increased working hours at weaning stage (hours/pig)	0.0056
Decreased working hours at fattening stage (hours/pig)	0.17
Prevalence of lung lesions at meat inspection (%)	5.0

Source: personal communications from experts at the Farm and Animal Health Association.

Most farms in Europe have quarantine units where purchased replacement gilts are held, either for a short period or until after farrowing or weaning (13). However, keeping the purchased groups of gilts from the existing sows for a longer time could increase their resistance to infections (30). The standard procedure in Sweden is to keep purchased groups of gilts in quarantine for 3 weeks and then place them in the same section as the existing sows once they have been inseminated (if not already pregnant at delivery). In scenario S2, it was assumed that purchased gilts were 7-weeks pregnant and were kept separated from the sows during gestation, farrowing and lactation. S2 was based on a Swedish study, where the results revealed that detection of pleurisy at slaughter decreased by 0.8–40.3% when keeping gilts separated from sows (22). Implementing S2 involves the establishment of a new facility to keep the gilts in quarantine for 9.5 weeks until the litter is weaned, an acclimatization strategy that has been proven to reduce the spread of bacteria from older parity sows to newly purchased gilts (30). The additional variables included in the partial budget analysis to model S2 are summarized in Table 3.

**Table 3:** Additional variables from the baseline farm model used in the partial budget model for strategy S2 (keeping purchased gilts separated from existing sows).

**Variables**	**Mean value**
Farm mortality (%)	0.9
Decreased working hours at fattening stage (hours/pig)	0.17
Yearly cost of building a separate space for gilts (SEK)*	124,250
Prevalence of lung lesion at meat inspection (%)	3.0

Source: personal communications from experts at the Farm & Animal Health Association.

^*^Calculations are available in Supplementary material 2.

Strategies S1 and S2 both involve important practices that can control the spread of infections causing lung lesions (21, 22). Calculations on the space required for the quarantine of the purchased gilts in S2 and for keeping them separated from the other sows on the farm are provided in Supplementary material 2.

### Stochastic partial budget analysis

Using partial budget analysis, a farm manager can evaluate whether a change in management or production practices will increase or decrease profit (14, 16). The method, however, does not determine profitability. It determines only the change in profitability, which is measured by the net benefit change as:


$$\begin{array}{l} \text{Net benefit change }=\text{Total benefit change }-\text{Total cost change} \end{array}$$

where, total benefit change includes costs saved and new revenues, while total cost change includes new costs and revenue foregone.

The stochastic partial budget analysis involves a budgeting approach using Monte Carlo simulations. To make the analysis stochastic, probabilities of occurrence need to be coupled to possible values of the key factors in a deterministic budget, thereby generating the probability distribution of possible budget outcomes (14, 19). The analysis in this study was performed using the Excel add-on @Risk (Palisade, Ithaca, NY) running 1,000 iterations.

In impact (sensitivity) analysis, several possible outcomes with a variety of input parameters are computed and subsequently displayed along with the probability to occur. This helps the decision-maker assess which risks to accept and which risks to avoid, allowing for optimal decision-making under uncertainty.

### Partial budget model on the effects of strategy S1 (avoiding mixing litters)

In the scenario that involved avoiding mixing litters after weaning (S1), the total amount of finishing pigs on the farm was divided into four different categories, as listed in Table 4. The breakdown components of net benefit change in this case comprised costs saved, new costs and revenue forgone.

**Table 4:** Categories of finishing pigs used in the partial budget model when either adopting or not adopting the strategy of avoiding mixing litters (S1).

**Categories of pigs**	**If not adopting S1 (I)**	**If adopting S1 (II)**
Number of pigs with no lung lesions at meat inspection (pigs A)	A(I)	A(II)
Number of pigs with lung lesions at meat inspection (pigs B)	B(I)	B(II)
Number of pigs with respiratory infections that die before slaughter (pigs C)	C(I)	C(II)
Number of foregone pigs* from not maximizing use of available space (pigs D)	D(I)	D(II)

^*^Foregone pigs = pigs that cannot be used in production because of sub-optimal use of space (i.e., uneven numbers of pigs in groups).

#### Costs saved

- Decreased costs of medicine, treatment and vet visits = cost per sick pig (SEK/slaughter pig) × number of sick pigs (with strategy S1 not adopted). It was assumed that the decrease in the number of sick pigs with the adoption of S1 was equal to half the change in the number of sick pigs B and all the changes in the number of pigs C, giving: 0.5 × (B(I) - B(II)) + (C(I) - C(II)).
- Decreased labor hours at the finishing stage = decreased working hours × labor cost per hour.
- Decreased cost of daily feed for foregone pigs = average days fed before slaughter × average daily intake × feed cost × number of pigs that cannot be raised because of non-maximized use of space [i.e., D(II)].
- Decreased cost of carcass disposal = unitary cost for carcass disposal × decreased number of pigs dying of respiratory disease before slaughter: [C(I) - C(II)].
- Decreased deduction costs for lung lesions at meat inspection = unitary deduction price × change in the number of pigs showing lung lesions at meat inspection: [A(I) - A(II)].

#### Revenue foregone

Decreased carcase sales = average slaughter weight with strategy S1 (kg) × meat sales (price/kg) × increased number of finishing pigs sent to slaughter because of strategy S1 [D(II)].

#### New costs

- Increased feed costs = average daily intake × feed cost × average days fed × increased number of pigs without lesions at meat inspection: [C(I) - C(II)].
- Increased labor input, for pigs C with respiratory infections that die before slaughter = average work hours per day (at farm level) × labor cost × (average production days – average production days for animal dead before slaughter).
- Increased labor hours at weaning = increased working hours × labor cost.

In scenario S1 of avoiding mixing litters there were no increased benefits, so:


$$\begin{array}{l} \text{Net changeS}1\;=\text{Costs saved}-\text{Revenue foregone }-\text{New costs}. \end{array}$$

### Partial budget model on the effects of strategy S2 (keeping purchased gilts separated)

In the scenario that involved keeping purchased gilts separated (S2), the total amount of finishing pigs on the farm was divided into three different categories, as listed in Table 5. The breakdown components for the cost and benefit analysis are displayed below.

**Table 5:** Categories of finishing pigs used in the partial budget model when either adopting or not adopting the strategy of keeping purchases gilts separated (S2).

**Categories of pigs**	**If not adopting S2 (III)**	**If adopting S2 (IV)**
Number of pigs with no lung lesions at meat inspection (pigs A)	A(III)	A(IV)
Number of pigs with lung lesions at meat inspection (pigs B)	B(III)	B(IV)
Number of pigs with respiratory infections that die before slaughter (pigs C)	C(III)	C(IV)

#### New revenue

- Increased carcase sales = average slaughter weight with strategy S2 (kg) × meat sales (price/kg) × increased slaughter pig number because of strategy S2.

#### Costs saved

- Decreased costs of medicine, treatment and vet visits = unitary cost per sick pig (SEK/pig) × number of sick pigs (with strategy S2 not adopted). The decreased number of sick pigs was assumed to equal half the change in the number of sick pigs B and all the change in the number of pigs C, giving: 0.5 × (*B(III) - B(IV)*) + (C(III) - C(IV)).
- Decreased labor hours at the finishing stage = decreased working hours × labor cost.
- Decreased cost of carcass disposal = unitary cost for carcass disposal × decreased number of pigs dying of respiratory diseases before slaughter: [C(III) – C(IV)].
- Decreased deduction costs for lung lesions at meat inspection = unitary deduction price × change in the number of pigs showing lung lesions at meat inspection.

#### New costs

- Increased feed cost = average increased daily intake × feed cost × average days fed × increased number of pigs without lung lesions at meat inspection: [C(III) - C(IV)].
- Increased labor input for pigs C, for decreased number of pigs with respiratory infections that die before slaughter = average work hours per day (at farm level) × labor cost × (average production days – average production days for animal dead before slaughter).
- Increased cost of a separate building for gilts.

In scenario S2 of keeping replacement gilts separated there were no foregone benefits, so:


$$\begin{array}{l} \text{Net changeS}2\;=\text{New revenue}+\text{Costs saved}-\text{New costs}. \end{array}$$

## Results

Deterministic results of partial budget analysis are reported in Tables 6, 7, while stochastic results are given in Table 8.

**Table 6:** Deterministic effects on net benefit change of adopting strategy S1 (avoiding mixing litters).

**Benefit change (SEK)**		**Cost change (SEK)**	
**New revenue**		**Revenue foregone**	
	0	Decreased carcase sales due to sub-optimal use of space	196,295
**Costs saved**		**New cost**	
Decreased costs of medicine, treatment and vet visits.	3,693	Increased feed costs	58,050
Decreased labor hours at finishing stage	59,826	Increased labor input for pigs with respiratory infections that die before slaughter	2,001
Decreased cost of daily feed for foregone pigs	157,317	Increased labor hours at weaning	2,005
Decreased cost of carcass disposal	44,994		
Decreased deduction costs for lung lesions at meat inspection	26,106		
**Subtotal**	**291,936**	**Subtotal**	**258,351**
**Net (benefit) change**		**33,585 SEK**	

**Table 7:** Deterministic effects on net benefit change of adopting strategy S2 (keeping purchased gilts separated).

**Revenue change (SEK)**		**Cost change (SEK)**	
**New revenue**		**Revenue foregone**	
Increased carcase sales	210,716		0
**Costs saved**		**New cost**	
Decreased costs of medicine, treatment and vet visits	4,114	Increased feed costs	56,634
Decreased labor hours at finishing stage	67,753	Increased labor input for pigs	2,001
Decreased cost of carcass disposal	43,897	Cost of building for gilts	124,250
Decreased deduction costs for lung lesions at meat inspection	29,565		
**Subtotal**	**356,045**	**Subtotal**	**182,885**
**Net (benefit) change**		**173,160 SEK**	

**Table 8:** Distribution of net benefit change of S1 and S2 from stochastic simulations.

**Net benefit change**	**Median**	**sd**	**5%**	**95%**
Net benefit change of S1	35,392	44,570	−44,823	102,573
Net benefit change of S2	171,124	382,189	111,627	237,716

### Effects of avoiding mixing litters (strategy S1)

The deterministic results of partial budget analysis (Table 6) revealed the main factors influencing the net benefit change incurred by adopting S1. The left part of Table 6 shows the benefit change due to strategy S1 (including increased revenues and reduced costs). There was no increased revenue when adopting S1. In terms of reduced costs, the largest change was in decreased costs of daily feed for foregone pigs (157,317 SEK), followed by decreased labor costs in the finishing phase (59,826 SEK). The subtotal for benefit change when adopting strategy S1 was 291,936 SEK. Cost changes due to strategy S1, which are shown in the right part of Table 6, included decreased revenues and increased costs. Decreased revenues derived from decreased carcase sales (196,295 SEK). Increased feed cost was the largest contributor to increased costs (58,050 SEK). The subtotal cost change was 258,351 SEK. Deducting the subtotal cost change from the subtotal benefit change gave an annual net benefit change of 33,585 SEK (with sd of 44,569 SEK, a median of 35,392, and 90% central range: −44,828; 102,573. See Table 8) and a per-animal net benefit change of 3.61 SEK. At the time of the analysis (6 July 2022), the exchange rate between Swedish Krona and Euro was 1 SEK = 0.093€.

**Figure 1:**
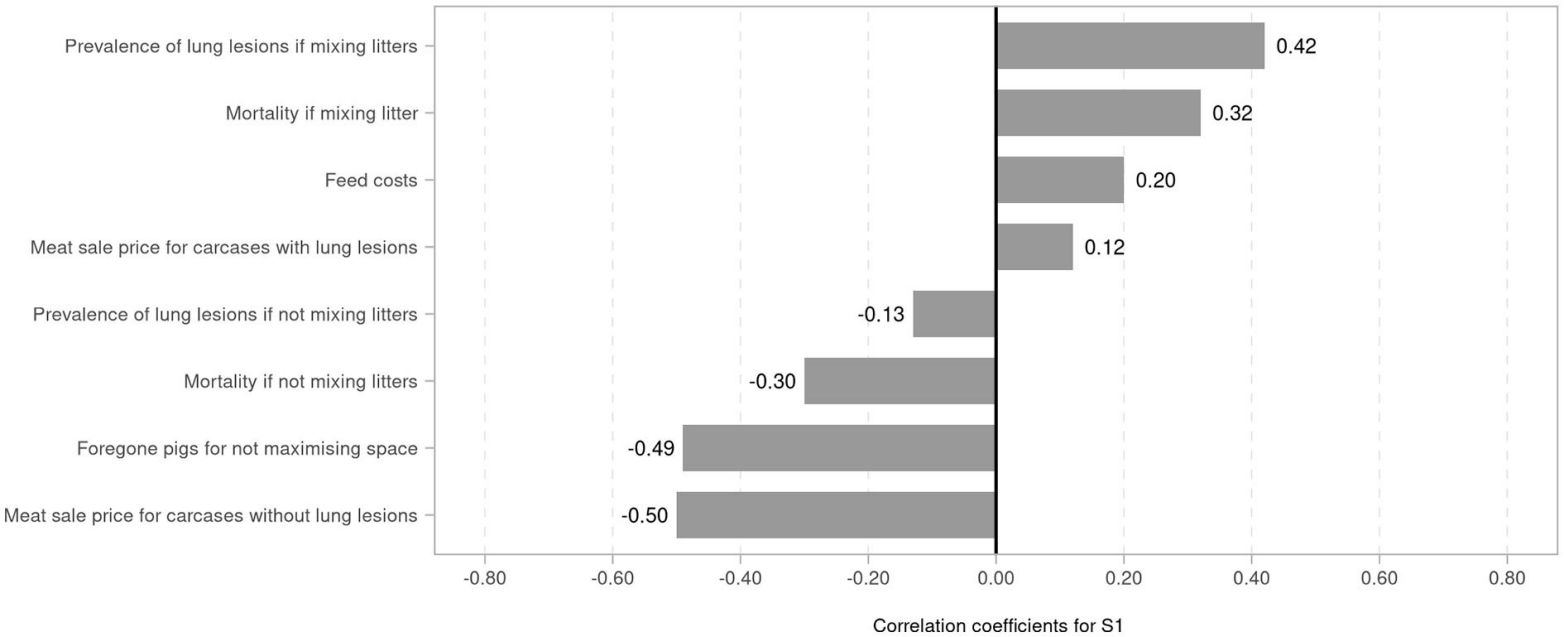
Tornado plot with correlation coefficients of drivers of net benefit change when avoiding mixing litters (strategy S1).

The results of the impact (sensitivity) analysis of strategy S1 are shown in Figure 1. The higher prevalence of lung lesions when mixing litters was the most influential factor in determining net benefit change, followed by the mortality rate if mixing litters (correlation coefficient 0.42 and 0.32, respectively). Feed cost was estimated to be positively correlated with net benefit change (0.20), and with a meat sale price for carcases with lung lesions (0.12). Meat sale price for carcases without lung lesions was strongly negatively correlated with net benefit change (−0.50), followed by foregone raised pigs (−0.49). Mortality and prevalence of lung lesions if not mixing litters were estimated to be negatively correlated with net benefit change (Figure 1).

### Effects of keeping purchased gilts separated (strategy S2)

Results for the partial budget analysis related to changes under strategy S2 are provided in Table 7. Increased carcase sales gave 210,716 SEK, which was the most influential variable in determining benefit change, followed by decreased labor costs in the finishing stage and decreased cost of carcase disposal (67,753 SEK and 43,897 SEK, respectively). However, this increase in benefits was offset by increased costs of 182,885 SEK due to increased feed cost (56,634 SEK), labor cost (2,001 SEK), and cost of a new building for gilts (124,250 SEK). The annual net benefit change was 173,160 SEK (with sd of 38,218 SEK, a median of 171,124, and a 90% central range: 111,627; 237,716. See Table 8) and the per-animal increase in net benefit under S2 was 18.61 SEK.

**Figure 2:**
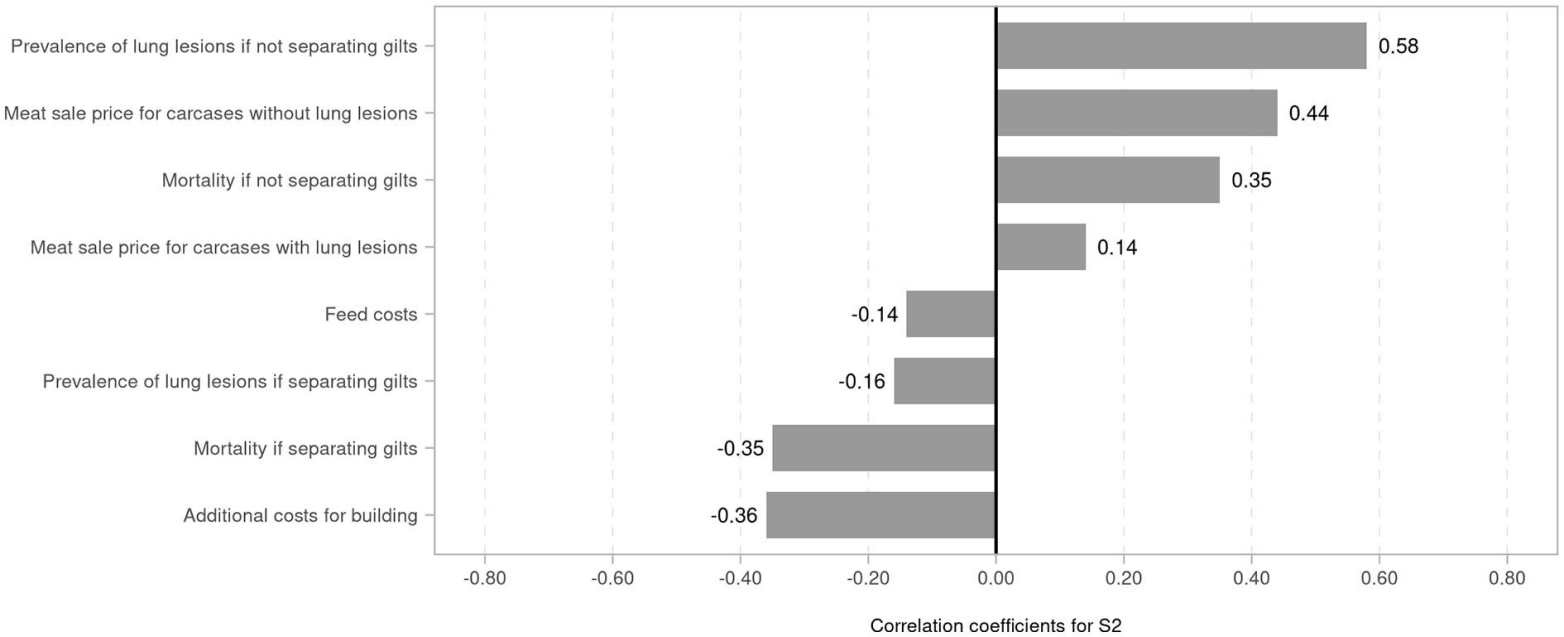
Tornado plot with correlation coefficients of drivers of net benefit change when keeping purchased gilts separated (strategy S2).

A tornado diagram with correlation coefficients of drivers of net benefit change under S2 shows that the prevalence of lung lesions when not separating gilts was the most influential factor, with a correlation coefficient of 0.58, followed by meat sale price without lung lesions (correlation coefficient 0.44) (Figure 2). The mortality rate when not separating gilts was positively correlated with net benefit change (0.35). The cost of a building for gilts was most strongly negatively correlated with net benefit change (−0.36), followed by mortality rate if separating gilts (−0.35). The prevalence of lung lesions if separating gilts and feed costs was estimated to be negatively correlated with net benefit change (Figure 2).

## Discussion

Using economic production data for an average Swedish farrow-to-finish pig farm with 355 sows, meat inspection data from 468,774 pig carcases derived from 53 different farms and expert opinions, this study explored the economic impact of adopting two strategies aimed at reducing the prevalence of lung lesions detected during meat inspections of finishing pigs. In both cases, the adoption of the strategy resulted in a positive change in net income.

Proper cleaning and vaccination programmes are performed more or less routinely on Swedish pig farms. The practices of mixing litters after weaning, to even out litter size, and keeping purchased gilts in quarantine for 3 weeks are also performed routinely. It is well-known that respiratory pathogens (e.g., Mhyo and App) are transmitted when pigs with differences in bacterial flora are placed next to each other or if there are too many pigs per unit floor area (30). However, the economic benefits of implementing strategies to control the spread of these respiratory pathogens have not been studied previously. This study assessed the costs associated with implementing two strategies [avoiding mixing litters after weaning and keeping the group intact until slaughter (S1) and keeping purchased pregnant gilts separated for a longer period (S2)], with both assumed to lead to a decreased spread of pathogens and thereby to more profitable pig production.

In both models, we adopted mean farm mortality of 1.80% and assumed it was normally distributed with 10% sd. The mean of farm mortality was derived from WinPig data for 2018. The WinPig dataset did not include the distribution and sd, so we consulted experts who had strong and long working experience on this perspective, and then we assumed it had a normal distribution with sd of 10%. We also tried models with 5% and 15% sd, which showed that the results were comparatively robust; therefore, we showed and discussed only the results with 10% sd in the manuscript. Please see the sensitivity analysis for both strategies with 5% and 15% sd in the Supplementary material.

The strategy of avoiding mixing litters after weaning (S1) has the advantage of being easy and straightforward to implement; however, it comes with the risk of a negative revenue (Table 8) related to the number of pigs that cannot be used in production because of sub-optimal use of space. Farmers need therefore to evaluate risks and benefits according to their own situation. On the other hand, the strategy of separating newly purchased breeding stock (S2) was found to be associated with higher revenue but had the disadvantage of requiring a high initial investment in building the extended quarantine facility and a separate farrowing unit unless already existing buildings could be uses to elongate the adaptive phase for the replacers. If not, this high initial investment cost brings additional financial risks, increased costs per sow and increased amortization costs. This study did not consider future uncertainty or market volatility and assumed that the purchased gilts in S2 were pregnant at delivery.

The baseline farm model was based on an average farrow-to-finish pig farm, a system that requires high inputs of capital and labor but has great market potential and flexibility since the producer has control of the entire production cycle. Despite the suggested measures being meant to be applied during the earlier production phases (i.e., gestation and growing), the stochastic partial budget analysis was based on the finishing phase of production, which has the greatest impact on the entire production cycle and the most frequent detection of respiratory diseases. Raising healthy piglets is a key factor for a healthy and high-yielding pig population, so strategies S1 and S2 could also be applied on farrow-to-feeder farms. However, such producers would not be equally incentivised to adopt the strategies, as the greatest economic benefits (e.g., reduction of lung lesions detected at meat inspection, heavier and better carcases, and thus better meat sale price) would be gained by finishing pig producers buying healthier piglets who ultimately have less lung lesions and thus higher revenue for carcass sales.

The two proposed measures can have a positive impact on animal welfare as well, as healthier animals thrive better. The interest in explaining animal welfare in terms of production economics is increasing, e.g., Alvåsen et al. (26) performed an economic analysis on animal welfare for nurse sows, and Henningsen et al. (20) analyzed the empirical relationship between animal welfare and the economic performance of Danish pig farms, and Ahmed et al. (19) investigated the effect of space allowance on animal welfare and profitability for cattle fatteners. Those studies found various degrees of positive relationships between animal welfare indicators and economic outcomes, indicating the advantages of describing and estimating the economic effects of animal health and welfare. Although stochastic partial budget analysis can be applied in a variety of decision-making situations for farmers (31–37), there are some limitations to this method. First, it is restricted to evaluating only two alternatives and one of the alternatives is related to current operations. This meant that we compared cost-benefit change between the baseline farm and S1 and that between the baseline farm and S2, but the results cannot be used directly used to compare S1 and S2. Second, only cost and benefit changes that are affected by the intended decision are considered in the partial budget, and not the external situation. If all areas affected by an intended change are not identified, the evaluation of the impacts of the change might be inaccurate. Third, the results obtained are estimates that are only as accurate as the original data used, and erroneous or inaccurate data can lead to biased results. Although stochastic partial budget analysis has been improved in this regard, caution is needed when using prior information for the analysis. In this context, it is a strength that this study used data from real studies (27, 37) as well as knowledge from experienced farm advisors. Even though the assumptions adopted in this study were likely to be reliable, there was a large variation in most input variables on commercial farms. This led to a large variation in the outcomes (Table 7), which requires extra caution when translating the results into operational activities. Additionally, the results of this study are mainly valid for the Swedish pig production system, which is known for having one of the highest animal welfare standards in the world (e.g., tail docking is banned, sows cannot be created during farrowing and suckling, and there is more space per pig in pens). Therefore, countries with lower animal welfare standards (e.g., where large groups of pigs are kept together, piglets are weaned at earlier age and sows are kept in crates during gestation) could be assumed to gain even higher economic benefits due to greater improvements in standards from adopting the two strategies analyzed in this study. If the study is replicated elsewhere, local production conditions must be taken into account.

There are a few additional limitations in this study that need to be addressed. First, there has been limited research on the economic benefits of implementing strategies to reduce the prevalence of respiratory diseases in pigs, which limited the input variables available for the analysis. This was also noted by Ahmed et al. (19), who experienced issues when estimating the exact health benefits in terms of production and profits of increased space allowance for cattle fatteners. Second, estimating the economic impact of strategies to reduce the prevalence of lung lesions is challenging, since co-infections of respiratory and non-respiratory (e.g., gastrointestinal) pathogens can occur simultaneously and it is impossible to separate their individual burden of disease. Third, if respiratory disease occurs during the growing phase or even earlier, there is a chance that any lung lesions will heal and not be detected during meat inspection (38). This means that the prevalence of lung lesions on-farm may be higher than recorded in meat inspections. Fourth, we did not explicitly consider feed conversion efficiency between pigs with and without lung lesions. However, we accounted for it indirectly in the model, by assuming that healthier pigs—because of S1 or S2—would be heavier at slaughter and have a better carcase score.

Potential economic benefits are the major incentives that could motivate producers to apply any preventive or suppressive measures. This study showed that by adopting the strategies of avoiding mixing litters and extending the quarantine period of purchased pregnant gilts, finishing pig producers can lower the prevalence of lung lesions recorded at slaughter and potentially increase the net economic benefit of production. However, Alvåsen et al. (39) point out the risk with assessing direct economic effects of animal health and welfare aspects, as it consists of both monetary and ethical values. The dilemma is that ethical values cannot be included in economic calculations and estimated in monetary terms. This also applied to the present study, where we identified a need for research covering ethical aspects of measuring animal welfare in economic terms. We also identified a need for further research on the economic aspects of animal health in pig production.

## Conclusion

This study provided useful insights into the economic impact of two different strategies for reducing the prevalence of lung lesions in finishing pigs. Both strategies were found to be potentially economically sustainable under the assumed conditions. Impact analysis of the simulation models for the two strategies revealed that a higher prevalence of lung lesions if not adopting one of the strategies was the main factor determining the net benefit change on-farm. With the increasing prevalence of lung lesions (10), adopting an effective strategy to decrease respiratory infections will become more relevant and more economically beneficial. The results presented here can be used to support pig producers in choosing cost-effective management strategies to reduce the prevalence of lung lesions at slaughter by improving animal health and welfare.

## Data availability statement

The raw data cannot be provided as it is covered by GDPR. All data required to replicate this work is provided (in aggregated form) in article tables and Supplementary material.

## Author contributions

JJ: conceptualization, data acquisition, and writing—original draft, review and editing. WH: conceptualization, methodology and data analysis, and writing—review and editing. C-JE, IE, and AR: conceptualization, data acquisition, and review and editing. AC: conceptualization, data acquisition, writing—review and editing, funding acquisition, and project administration. All authors contributed to the article and approved the submitted version.

## Funding

This work was funded by the Swedish Research Council FORMAS (Grant No. 2017-00593).

## Conflict of interest

The authors declare that the research was conducted in the absence of any commercial or financial relationships that could be construed as a potential conflict of interest.

## Publisher's note

All claims expressed in this article are solely those of the authors and do not necessarily represent those of their affiliated organizations, or those of the publisher, the editors and the reviewers. Any product that may be evaluated in this article, or claim that may be made by its manufacturer, is not guaranteed or endorsed by the publisher.
