# Supplementary material for: Stochastic partial budget analysis of strategies to reduce the prevalence of lung lesions in finishing pigs at slaughter
Source: Front Vet Sci. 2022 Oct 14;9:957975. doi: 10.3389/fvets.2022.957975 (PMC9614246; doi:10.3389/fvets.2022.957975)
Supplement: Supplementary file 1 [file Data_Sheet_1.PDF]

Supplementary material 1 – Key figures in farrow-to-finish pig production

A. Key figures in sows and piglet production (gestation, farrowing, weaning) on a yearly basis. (source: WinPig®, 2018)

**Variable**	**Value**
Labour, employees	14.39 h/sow[a]
Cost of labour, employee	3 958 SEK/sow
Buildings, interest, depreciation	5.352 SEK/sow
Electricity, water, heating	465 SEK/sow
Purchased feed cost[b]	8.344 SEK/sow
Veterinarian, iron supplement	963.06 SEK/sow
WinPig® supervisory services	60.17 SEK/ sow
Straw	360.56 SEK/sow
Boar, insemination	736 SEK/sow
Recruitment, 62.5%	641 SEK/sow
Other costs[c]	152.18 SEK/sow
Piglets, 26.7/sow	17.623 SEK/sow
Meat, removed sows, 52.7%	479 SEK/sow
Stable dung, m^3	160 SEK/sow
Subsidy	1 000 SEK/sow

[a] equals to 41 minutes per produced piglet
[b] Feed during gestation and the suckling phase for the sow, and feed during piglet and grower phase
[c] Insurances, carcass destruction, certifications, computer, education etc.

B. Key figures in finishing pig production (growers until finishing phase) on a yearly basis. (source: WinPig®, 2018).

**Variable**	**Value**
Labour, employees	0.25 h/finishing pig
Cost of labour, employees	68.86 SEK/finishing pig
Buildings, interest, depreciation	176.69 SEK/finishing pig
Electricity, water, heating	19.17 SEK/finishing pig
Feed cost	634.09 SEK/finishing pig
Veterinarian, medicine and health costs	2.75 SEK/finishing pig
WinPig® supervisory services	2.14 SEK/finishing pig
Straw	4.18 SEK/finishing pig
Other costs[a]	16.14 SEK/finishing pig
Reduction of slaughter remarks[b]	20 SEK/remark of lung lesions
Meat sale, 16.3 SEK/kg	1506.1 SEK/finishing pig
Stable dung, 19 SEK/m^3	13.3 SEK/finishing pig

[a] Insurances, carcass destruction, certifications, computer, education etc.
[b] The value of the reductions differs between abattoirs
