# Supplementary material for: Stochastic partial budget analysis of strategies to reduce the prevalence of lung lesions in finishing pigs at slaughter
Source: Front Vet Sci. 2022 Oct 14;9:957975. doi: 10.3389/fvets.2022.957975 (PMC9614246; doi:10.3389/fvets.2022.957975)
Supplement: Supplementary file 2 [file Data_Sheet_2.PDF]

Supplementary material 2 - Underlying calculations for strategy S2 (extended quarantine and individually ventilated farrowing units)

Data were taken from baseline farm models (2018) regarding herd size and production level: 355 productive sows x 26.7 produced piglets per sow and year = 9,479 finishing pig produced per year.

Part 1: Extended quarantine and cost of the extra space required

Assuming the 355 productive sows (including pregnant gilts) are divided into 11 groups of 32 sows, the space requirements would be:

- 9 groups simultaneously in gestation units (including dry sows)
- 3 farrowing units
- 4 finishing units

Required breeding stock: 8 gilts per group and farrowing

For purchased gilts (7 weeks pregnant) to be kept separated from the other sows in the herd before farrowing, the required extended quarantine is 9.5 weeks.

<u>Calculations for the new building</u>

Required space: 2 units x 8 gilts require 60 m^2

Costs:

- flooring, walls and roof = 4,500 SEK/m^2
- interior for 2 x 8 gilts = 67,000 SEK (including mounting)
- ventilation for 2 x 8 gilts = 45,000 SEK (including mounting)
- wet feed for 2 x 8 gilts = 2,250 SEK per pipe → 36,000 SEK

If a new group of gilts arrives every other week, 5 sections with 8 gilts per section are required.

We assumed that one gilt section for quarantine already existed on the farm, so 4 additional sections with 8 places each are required.

Cost of new building:

Building: 2 x 60 m^2 x 4,500 SEK/m^2 = 540,000 SEK

Interior: 2 x 67,000 = 134,000 SEK

Ventilation: 2 x 45,000 = 90,000 SEK

Wet feed: 32 places x 2,250 SEK/pipe = 72,000 SEK

<u>**TOTAL: 836,000 SEK**</u>

Part 2: Farrowing units with individually ventilated sections

For 11 groups of sows, 3 farrowing units are needed.

Cost of dividing walls between the original herd sows and recruitment gilts: 50,000 SEK per section (including labour needed)

Cost of separate ventilation: 50,000 SEK per section (including mounting)

- ⇨ extra costs of approximately 100,000 SEK per section x 3 = 300,000 SEK in total

Additional costs are higher labour costs when opening and closing more doors and more time-consuming traffic in the aisles. Expected benefits from healthier pigs and less time spent on treatment for sick pigs with lung lesions, in the best of worlds, would compensate for these additional costs.

- ⇨ The overall increased cost per sow and year is expected to be 350 SEK.
