# Supplementary material for: Stochastic partial budget analysis of strategies to reduce the prevalence of lung lesions in finishing pigs at slaughter
Source: Front Vet Sci. 2022 Oct 14;9:957975. doi: 10.3389/fvets.2022.957975 (PMC9614246; doi:10.3389/fvets.2022.957975)
Supplement: Supplementary file 3 [file Data_Sheet_3.pdf]

Supplementary material 3 - Sensitivity analysis on the impact of variance of mean farm mortality

Sensitivity analysis for both strategies assuming the standard devition (sd) of the mean farm mortality to be 5% and 15%, respectively.

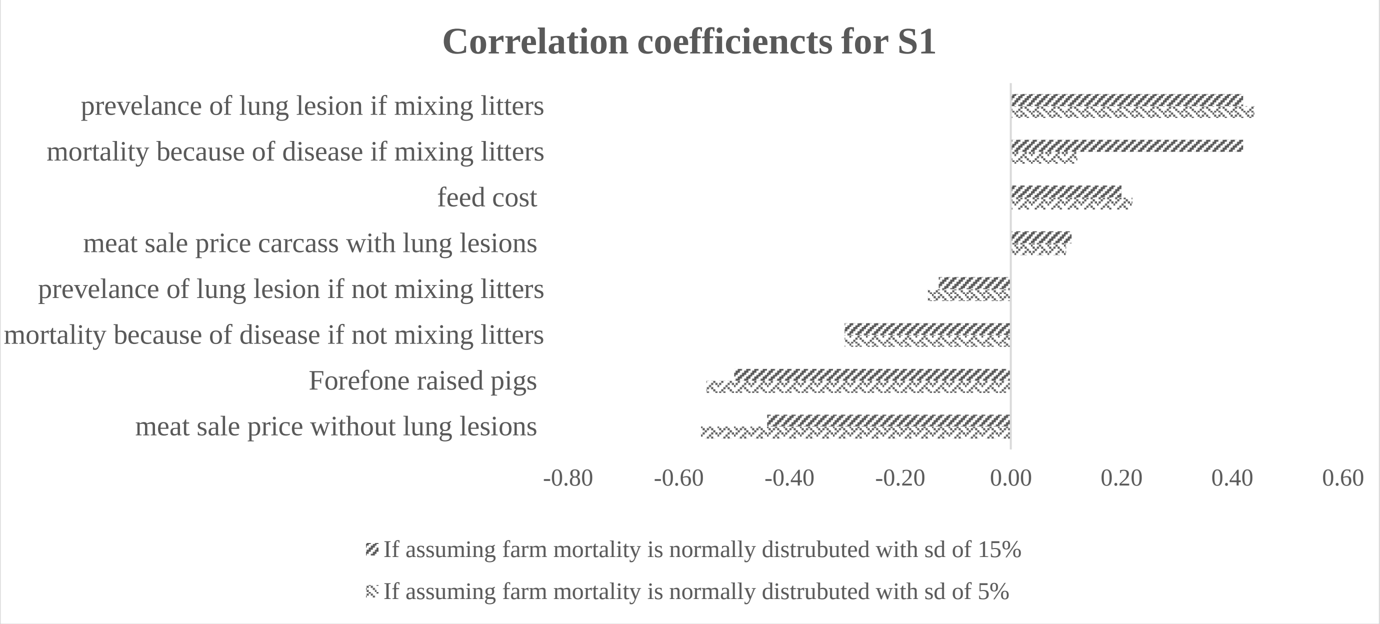

Figure s1. Correlation coefficients for SM1 if assuming farm mortality is normally distributed with sd of 5% and 15%

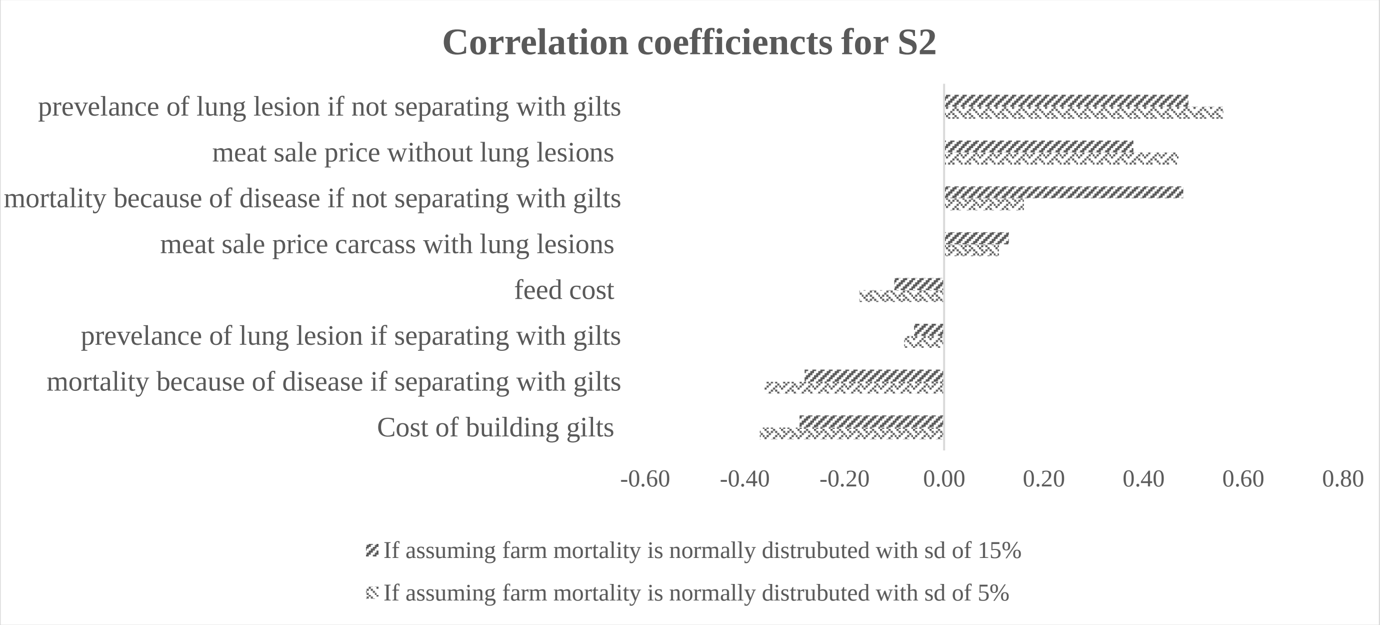

Figure s2. Correlation coefficients for SM2 if assuming farm mortality is normally distributed with sd of 5% and 15%
